# *METTL14* alleviates heat stress in Hu sheep involves enhancing fatty acid oxidation while reducing lipid deposition

**DOI:** 10.3389/fvets.2025.1732947

**Published:** 2026-01-19

**Authors:** Bowen Chen, Chao Yuan, Tingting Guo, Lixia Sun, Jianbin Liu, Zengkui Lu

**Affiliations:** 1 Key Laboratory of Animal Genetics and Breeding on the Tibetan Plateau, Ministry of Agriculture and Rural Affairs, Lanzhou Institute of Husbandry and Pharmaceutical Sciences, Chinese Academy of Agricultural Sciences, Lanzhou, China; 2 Sheep Breeding Engineering Technology Research Center of Chinese Academy of Agricultural Sciences, Lanzhou, China

**Keywords:** heat stress, lipid deposition, m6A methylation, *METTL14*, sheep

## Abstract

**Objective:**

Heat stress significantly compromises sheep production performance, product quality, and overall health, leading to increased management costs and reduced profitability. Previous studies from our group demonstrated that the m6A methyltransferase gene *METTL14* is involved in both the heat stress response and the regulation of lipid metabolism in Hu sheep, suggesting a potential role in mediating heat stress through hepatic metabolic control. However, the specific mechanisms by which *METTL14* regulates heat stress and lipid metabolism, as well as the functional linkage between these processes, remain poorly understood

**Methods:**

We first established heat stress (HS), lipid deposition (LD), and lipid deposition heat stress (LDHS) models in Hu sheep hepatocytes and adipocytes. By interfering with and overexpressing the *METTL14* in these models, techniques such as qRT-PCR, immunofluorescence, RNA-seq, and LC-MS were employed

**Results:**

We found that *METTL14* contributes to the heat stress response under heat stress, suppresses the expression of heat shock-related genes, and significantly modulates lipid metabolism pathways. Under combined conditions of lipid accumulation and heat stress, *METTL14* participated in the lipid deposition process and downregulated the expression of associated genes. Furthermore, overexpression of *METTL14* under these conditions increased m6A methylation levels, downregulated heat shock genes (*HSP60*, *HSP70*, *HSP110*) and key lipogenic genes (*FABP4, PPARγ, Accα*). Notably, elevated expression of MTTP enhanced triglyceride export, ultimately reducing intracellular triglyceride content.

**Conclusion:**

In summary, this study unveils a novel mechanism through which *METTL14* mitigates heat stress in Hu sheep-by promoting fatty acid oxidation and attenuating lipid deposition.

## 1 Introduction

Ambient temperature is the predominant ecological factor influencing Hu sheep production. With the rapid development of intensive high-density farming systems and the increasing frequency of extreme heat events, heat stress has emerged as a critical challenge affecting Hu sheep. Heat stress not only severely impairs production performance (1–4), product quality (5), and health indices but also elevates management costs and reduces profitability (6, 7), necessitating urgent investigation into the molecular mechanisms underlying animal’s response to thermal stress.

m6A (N6-methyladenosine), a prevalent RNA modification, regulates gene expression, RNA stability, and translational efficiency (8–10). Studies demonstrate that heat stress significantly alters global m6A modification levels in animal cells (9). The dynamic equilibrium of m6A modifications is governed by “writers” METTL3 (methyltransferase-like protein 3) and METTL14 (methyltransferase-like protein 14), “erasers” FTO (fat mass and obesity associated protein) and Alk B homologue 5 (ALKBK5), “readers” YTHDF1 (YTH domain family proteins 1) and YTHDF2, whose activities or expression levels may shift under thermal stress (11, 12). Notably, while m6A methyltransferases and demethylases maintain subcellular localization during heat stress in mouse embryonic fibroblasts, YTHDF2 translocate from cytoplasm to nucleus under thermal challenge (13). Heat-induced m6A modifications correlate with altered gene expression patterns (12, 14), offering novel insights into thermal adaptation mechanisms. For instance, m6A modifications may regulate heat shock protein (HSP) gene expression (e.g., *HSP60*, *HSP70*, *HSP90*, *HSP110*) to enhance cellular thermotolerance (15), while concurrently modulating antioxidant, immune, and lipid metabolism genes to maintain physiological homeostasis (16, 17). Porcine studies reveal that hyperthermia upregulates hepatic *HSP27* and adipose *HSP70* expression while activating lipid metabolism genes (*ACACA*, *FASN*, *DGAT1*, *PPARγ*, *SREBP-1c*, and *FABP4*) in abdominal fat (18). Recent findings indicate that m6A regulators *METTL3* and *FTO* modulate heat shock gene expression through m6A-dependent mechanisms in Hu sheep hepatocytes and adipocytes (19), though *METTL14’s* regulatory role remains enigmatic.

Previous studies suggest m6A methylation influences Hu sheep’s thermal stress response and lipid metabolism, potentially mediating heat stress adaptation through hepatic lipid regulation (11, 20). Chen et al. further observed significant *METTL14* upregulation in heat-stressed and lipid deposition Hu sheep primary hepatocytes and preadipocytes (12, 21), yet its mechanistic interplay between lipid metabolism and heat stress regulation remains unresolved. This study employs *in vitro* models of Hu sheep primary hepatocytes and preadipocytes subjected to heat stress, lipid deposition, and lipid deposition heat stress conditions. Because the process of m6A methylation modification regulating heat stress is related to lipid metabolism, the study used preadipocytes as a further control to verify and fully explain the results of hepatocytes. Through *METTL14* knockdown/overexpression combined with quantitative real-time polymerase chain reaction (qRT-PCR) and multi-omics sequencing, we aim to decipher *METTL14’s* molecular mechanisms in regulating heat stress via lipid metabolism pathways, thereby providing scientific guidance for Hu sheep production under thermal stress conditions.

## 2 Materials and methods

### 2.1 Cell culture and identification

Three one-day-old newborn healthy Hu sheep (1.5–3 kg, ♂) from Lanzhou Wanshan Plantation and Breeding Professional Cooperative were used in this study. The sheep primary hepatocytes and preadipocytes isolation and culture procedures were similar to that previously reported (22). Briefly, those sheep were anesthetized with isoflurane inhalation (Sigma-Aldrich, St. Louis, MO, USA), bloodletting, and slaughter. The obtained liver tissues from three sheep were taken as mixed samples, and then the tissues were cut into 1 × 1 mm^3^ tissue blocks, 5 mL 0.25% trypsin (Gibco, Carlsbad, CA, USA) and 0.1 mg/mL type IV collagenase (Sigma-Aldrich, St. Louis, MO, USA) in a ratio of 1:1 was used for digestion and incubated at 37 °C for 15 min. The cells were filtered using a 100 μm sieve and cultured at 37 °C in a 5% CO_2_ incubator. After periodic acid–Schiff staining for glycogen and assessment of alpha-fetoprotein (AFP) expression, the isolated cells were identified as hepatocytes using the detection of hepatocyte-specific markers (cytokeratin (CK)-18 and albumin). Cell purity was at least 95% based on CK-18, AFP, and albumin staining (22). Perirenal adipose tissue of three sheep were taken as mixed samples. The adipose tissue was cut into 1 × 1 mm^3^ tissue blocks and digested with 1 mg/mL type I collagenase (Sigma-Aldrich) at 37 °C for 60–90 min. The cells were sequentially filtered with a 100 μm and 70 μm cell sieve, and cultured in a 5% CO_2_ incubator at 37 °C. Oil red O staining showed that small lipid droplets had appeared in some adipocytes that had grown to monolayer confluence, indicating that the isolated preadipocytes had the ability to proliferate and differentiate (12). Those cultures were tested and confirmed to be negative for mycoplasma contamination before use.

### 2.2 Bodipy staining

The steps of bodipy staining of hepatocytes and preadipocytes were similar to that previously reported (19, 21). Briefly, cultured cells were fixed with 4% paraformaldehyde for 15 min and incubated with PBS (Solarbio, Beijing, China) containing 1 μg/mL bodipy 493/503 (CHEMEGEN, Shanghai, China) stain for 20 min, then imaged using a ZEISS LSM800 confocal laser scanning microscope (Munich, Germany, Plan APOCHROMAT 10x/0.45). Image processing was carried out with ZEN software.

### 2.3 Establishment of relevant cellular models

The method of heat stress (HS), lipid deposition (LD), and lipid deposition heat stress (LDHS) models of hepatocytes and preadipocytes is the same as in the reported article. The HS condition: hepatocytes 42 °C incubator for 1 h, preadipocytes 42 °C incubator for 2 d (12); the LD condition: hepatocytes were incubated wit 1.2 mM fatty acid solution [oleic acid (OA): palmitic acid (PA) = 2:1] in William’s Medium E (Gibco) containing 15% FBS for 24 h, preadipocytes were incubated with a cocktail of insulin (10 μg/mL, Sigma-Aldrich), dexamethasone (1 μM, Sigma-Aldrich), and 3-isobutyl-1-methylxanthine (0.5 mM, Sigma-Aldrich) in DMEM/F12 with 10% FBS for 2 d, followed by culture with DMEM/F12, 10% FBS, and insulin (10 μg/mL) for another 2 d (21). The medium was replaced with DMEM/F12 supplemented with 10% FBS for 2 d; LDHS condition: treatment of lipid deposition heat stress conditions corresponding to hepatocytes and adipocytes, respectively (19).

### 2.4 Lentiviral overexpression and RNAi constructs and infection of cells

Construction of the *METTL14* overexpression vector and lentiviral packaging: the *METTL14* target fragment was cloned into the LV5-NC vector (EF-1a/GFP&Puro), using primers detailed in Supplementary Table S1. The recombinant plasmid was verified by sequencing and then produced in large-scale preparation. After lentiviral packaging, the viral titer was measured.

Construction of the *METTL14* interference vector and lentiviral packaging: short hairpin RNA (shRNA) sequences were designed to target *METTL14*, with the specific target sequence 5′- TTGGCCGACAGATTTGAAGAA’. This synthesized shRNA was inserted into the LV3-shNC vector (H1/GFP&Puro) via restriction enzyme digestion and ligation. The lentiviral particles were then packaged, and their titer was determined. All lentiviruses, including the corresponding negative controls (LV5-NC and LV3-NC), were synthesized and packaged by Shanghai GenePharma Co., Ltd.

For overexpression and interference experiments, primary hepatocytes and preadipocytes were washed with PBS and then transfected with *METTL14* overexpression (M14-OE), *METTL14* shRNA (M14-sR), or LV5-NC and LV3-NC lentivirus constructs for 72 h. Cells were isolated, and transfection efficiency was confirmed by qRT-PCR (*n* = 3, three technical repetitions were performed for each sample).

### 2.5 mRNA m6A methylation quantification

The method of relative mRNA m6A methylation was quantified using a EpiQuik mRNA m6A methylation quantification kit (Epigentek, St. Louis, MO, USA) and similar to that previously reported (19, 21). Briefly, total RNA was extracted from primary hepatocytes and preadipocytes. The corresponding binding solution, negative control, positive control, and RNA were then added to a 96-well plate for incubation at 37 °C for 90 min. Following a washing step, capture and detection antibodies were applied. An enhancer solution and developer solution were subsequently added. Absorbance was measured at 450 nm using a microplate reader. The percentage of m6A in total RNA was calculated using m6A % $=\frac{(\text{Sample}\;\mathrm{OD}-\mathrm{NC}\;\mathrm{OD})÷S}{(\mathrm{PC}\;\mathrm{OD}-\mathrm{NC}\;\mathrm{OD})÷P}$ , S is the amount of input sample RNA (ng); P is the amount of PC input (ng); PC was positive control, NC was negative control (*n* = 3, three technical repetitions were performed for each sample).

### 2.6 Detection of triglyceride (TG) content

TG content of primary hepatocytes and preadipocytes were determined according to the instructions of the TG kits (ZHONGSHENG, Beijing, China). A 4 μL sample or standard (*n* = 3) was added to 300 μL of R1 reagent and mixed thoroughly before incubation at 37 °C for 5 min. Next, 50 μL of R2 reagent was introduced, and the mixture was incubated again at 37 °C for 5 min. The absorbance at 500 nm was measured for both standards and samples using a microplate reader, with a reagent blank tube used for zero calibration. The triglyceride concentration (mmol/L) was calculated as (A sample / A standard) × Standard concentration, where the standard concentration is specified on the reagent kit label.

### 2.7 Immunofluorescence assay

Cultured hepatocytes were fixed with 4% paraformaldehyde for 15 min, washed with PBS, permeabilized with 0.3% Triton X-100 (Sigma-Aldrich, St. Louis, MO, USA) for 10 min, washed with PBS, and blocked with PBS containing 5% FBS and 0.3% TritonX-100 for 1 h. Thereafter, Primary antibodies against *METTL14* (Proteintech, Rosemont, IL, USA) and YTHDC2 (Proteintech) were applied overnight at 4 °C. After washing, cells were incubated with the goat anti-rabbit IgG conjugated with Alexa Fluor® 594 (Invitrogen, Waltham, MA, USA) or goat anti-mouse IgG conjugated with Alexa Fluor®488 (Invitrogen) for 1 h, followed by DAPI (Solarbio) nuclear staining. Imaging was performed using a ZEISS LSM800 confocal microscope (Munich, Germany, Plan APOCHROMAT 10x/0.45) and analyzed with ZEN software.

### 2.8 RNA sequencing (RNA-seq) data and analysis

The methods and steps of the RNA-seq data analysis were similar to that previously reported (19, 21). Briefly, the total RNA of primary hepatocytes was extracted with TRIzol reagent kit (Invitrogen, Carlsbad, CA, USA) according to the manufacturer’s protocol. Then the libraries (Illumina Novaseq6000 platform) were constructed using VAHTS Universal V6 RNA-seq Library Prep Kit after RNA quality assessed on an Agilent 2,100 Bioanalyzer. The OE Biotech Co., Ltd. (Shanghai, China) finished the transcriptome sequencing and analysis. Raw reads of fastq format were firstly processed using fastp (23). The clean reads were mapped to the reference genome using HISAT2 (24). Fragments Per Kilobase of exon model per Million mapped fragments (FPKM) (25) of each gene was calculated and the read counts of each gene were obtained by HTSeq-count (26). Then, differential expression genes (DEGs) between two groups were performed using DESeq2 (27). Genes/transcripts with *p* < 0.05 and |log-fold change| ≥ 1 were considered DEGs. GO and KEGG pathway enrichment analyses of the DEGs were performed using R based on hypergeometric distribution. GO terms and KEGG pathways with *p* < 0.05 were considered significantly enriched.

### 2.9 Ultra-high performance liquid chromatography-mass spectrometry (LC–MS) analysis

The methods and steps of LC–MS analysis were similar to that previously reported (19, 21). Briefly, the extracted lipid from primary hepatocytes by using 600 μL chloroform: methanol (2:1, v/v) were stored at −20 °C prior to LC–MS analysis. The Shanghai Luming biological technology co., LTD (Shanghai, China) finished the metabolomic data analysis. The LC system was performed using an ExionLC™ System. The temperature of the autosampler and oven were set at 4 °C and 55 °C, respectively. The positive and negative data were combined to get a combine data which was imported into R ropls package. Differential metabolites were further used to for KEGG pathway^1^ enrichment analysis.

### 2.10 Joint analysis of transcriptomic and metabolomic data

The method of joint analysis was similar to that previously reported (19, 21). Briefly, we used the Hmisc package in R1 to calculate pearson correlation coefficients between the differential metabolites and DEGs via pairwise comparison. DEGs and differential metabolites with a threshold of |r| > 0.7 and *p* < 0.05 were considered significantly correlated and were subjected to conjoint biological annotation using the KEGG database. The results visualized with the OmicShare tool, an online platform for data analysis^2^.

### 2.11 qRT-PCR and statistical analysis

Th reverse transcription, qRT-PCR and statistical analysis method of primary hepatocytes and preadipocytes was similar to that previously reported (19, 21). Briefly, we used a TransGen Biotech reverse transcription kit (Transgen, Beijing, China, refer to the instructions for specific methods) to reverse-transcribe extracted RNA. qRT-PCR was performed in 20 μL volumes per the manufacturer’s protocol (TransStar Tip Green qPCR SuperMix, Transgen) on a Bio-Rad C1000 Thermal Cycler. *β-actin* was used as a reference gene to normalize gene expression. The primer sequences used for qRT-PCR refer to (12, 21). Statistical analyses were performed using one-way ANOVA by SPSS 22 software. The results were displayed as mean ± SD by GraphPad Prism 8 software.

## 3 Results

### 3.1 *METTL14* is highly expressed in primary hepatocytes following HS and is associated with HS progression

**Figure 1:**
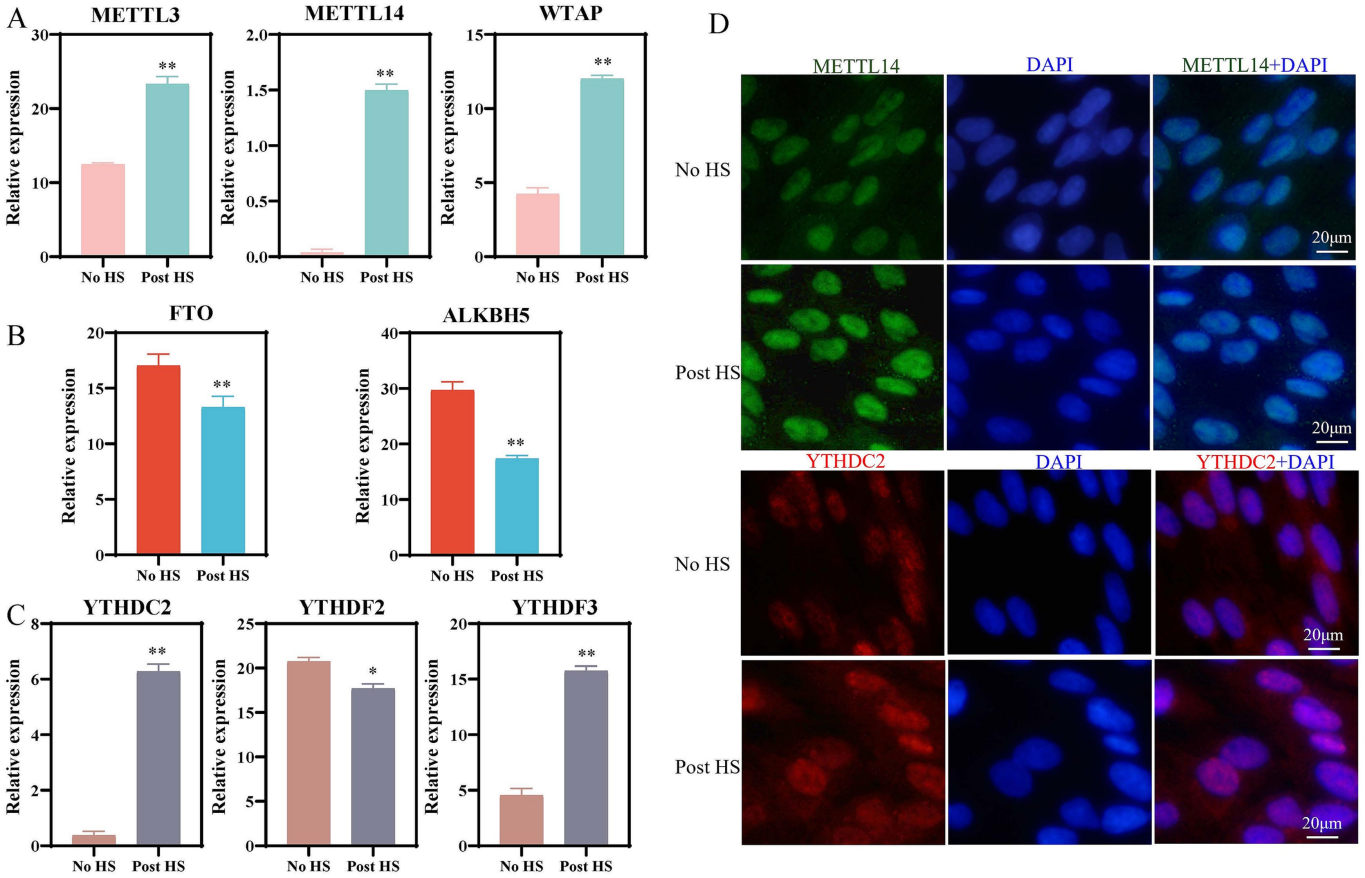
Changes in expression of methylation-related genes and immunofluorescence assays with and without HS in primary hepatocytes. **(A)** Expression of *METTL3*, *METTL14,* and *WTAP* in the RNA-seq of primary hepatocytes after HS; **(B)** Expression of *FTO* and *ALKBH5* in the RNA-seq of primary hepatocytes after HS; **(C)** Expression of *YTHDC2*, *YTHDF2* and *YTHDF3* in the RNA-seq of primary hepatocytes after HS; **(D)** Immunofluorescence assays of METTL14 and YTHDC2 with no HS and post HS. *p < 0.05, ** *p* < 0.01.

Our previous study demonstrated significant upregulation of both *METTL14* mRNA and protein expression in the liver tissue of heat-stressed Hu sheep (11). To further examine *METTL14* expression under heat stress, primary hepatocytes isolated from Hu sheep were subjected to 42 °C (No HS: normally cultured cells with no heat stress treatment, Post HS: normally cultured cells with 42 °C heat stress treatment). Transcriptome sequencing analysis confirmed a significant increase in *METTL14* expression under HS conditions (Figure 1A), which was consistent with qRT-PCR validation. Transcriptomic profiling also revealed markedly elevated expression of the m6A methyltransferases *METTL3* and *WTAP* (Figure 1A), whereas the demethylases *FTO* and *ALKBH5* were significantly downregulated (Figure 1B). Among m6A reader proteins, expression of *YTHDC2* and *YTHDF3* was significantly increased, while *YTHDF2* expression decreased (Figure 1C). Indirect immunofluorescence staining in heat-stressed hepatocytes indicated that *METTL14* were predominantly localized in the nucleus. Following heat stress, YTHDF2 translocated from the cytoplasm to the nucleus (12), suggesting HS-induced enhancement of its transcriptional activity. YTHDC2 was detected in both nuclear and cytoplasmic compartments (Figure 1D). These findings align with observations in heat-stressed preadipocytes, where *METTL14* expression and m6A methylation levels were also significantly elevated (12). Collectively, these results indicate that *METTL14* is highly expressed in primary hepatocytes after HS and is closely associated with the progression of heat stress.

### 3.2 *METTL14* suppresses the expression of heat shock genes and is associated with lipid metabolism pathways

To investigate the role of *METTL14* in the heat stress response, we first evaluated the efficiency of lentivirus-mediated knockdown and overexpression of *METTL14.* The results of qRT-PCR showed that *METTL14* expression was significantly increased following infection of primary hepatocytes with a lentiviral *METTL14* overexpression construct. Conversely, infection with a lentiviral *METTL14* RNAi construct led to significantly decreased *METTL14* expression (Supplementary Figure S1). The results of *METTL14* lentivirus infections of preadipocytes were similar.

**Figure 2:**
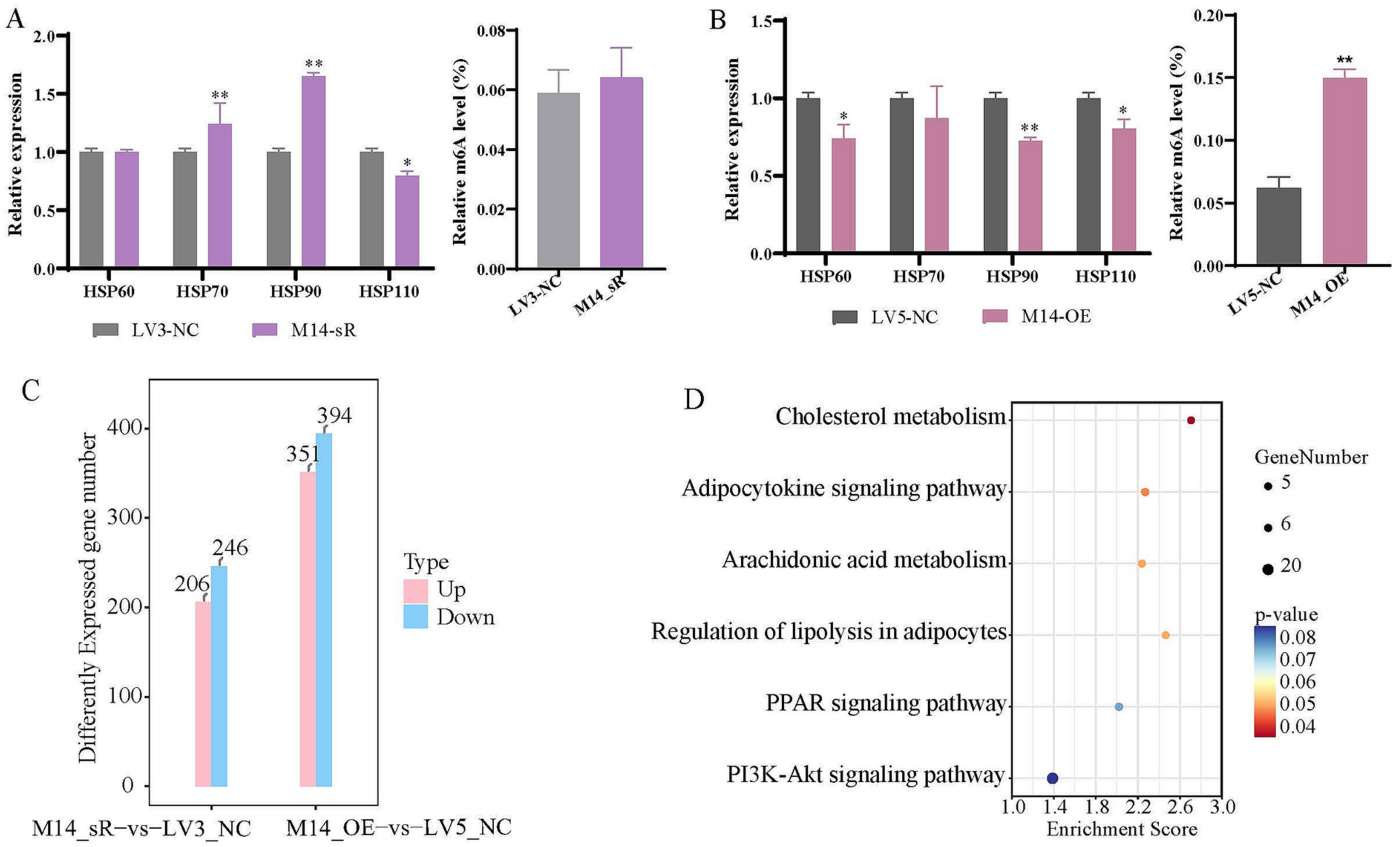
*METTL14* suppresses the expression of heat shock genes and is enriched in pathways associated with lipid metabolism. **(A)** Detection of the relative expression levels of heat shock-related genes and mRNA m6A methylation level for the M14_sR vs. LV3_NC group; **(B)** detection of the relative expression levels of heat shock-related genes and mRNA m6A methylation level for the M14_OE vs. LV5_NC group; **(C)** histogram of the number of DEGs for the M14_sR vs. LV3_NC group and M14_OE vs. LV5_NC group; **(D)** bubble diagram of the KEGG pathway enrichment analysis of DEGs in the M14_OE vs. LV5_NC group. * *p* < 0.05, **p < 0.01.

Compared with LV3_NC, expression levels of *HSP70* and *HSP90* were significantly increased after *METTL14* interference (Figure 2A); by contrast, expression levels of *HSP60*, *HSP90*, and *HSP110* significantly decreased under *METTL14* overexpression (Figure 2B), indicating that the overexpression of *METTL14* significantly inhibited the expression of heat stress relative genes, and the m6A methylation level significantly increased (Figure 2B). Similarly, in preadipocytes, the expression of *HSP60*, *HSP70* and *HSP110* significantly decreased (Supplementary Figures S2A,B), while the m6A methylation level significantly increased, following *METTL14* overexpression (Supplementary Figure S2B). These results indicated that overexpression of *METTL14* significantly suppressed heat stress gene expression in an m6A-dependent manner. To further understand the changes in signaling pathways involved in *METTL14* interference and overexpression, we compared RNA-seq results in primary hepatocytes under *METTL14* interference versus overexpression. Compared with negative control, 206 differentially upregulated genes and 264 differentially downregulated genes were screened in the *METTL14* interference group, while 351 differentially upregulated genes and 394 differentially downregulated genes were screened in the *METTL14* overexpression group (Figure 2C). The results of KEGG enrichment analysis showed these DEGs were significantly enriched in the cholesterol metabolism, adipocytokine signaling pathway and arachidonic acid metabolism pathways for the M14_OE vs. NC group (Figure 2D).

### 3.3 *METTL14* participates in the lipid deposition process and downregulates the expression of genes associated with lipid accumulation

**Figure 3:**
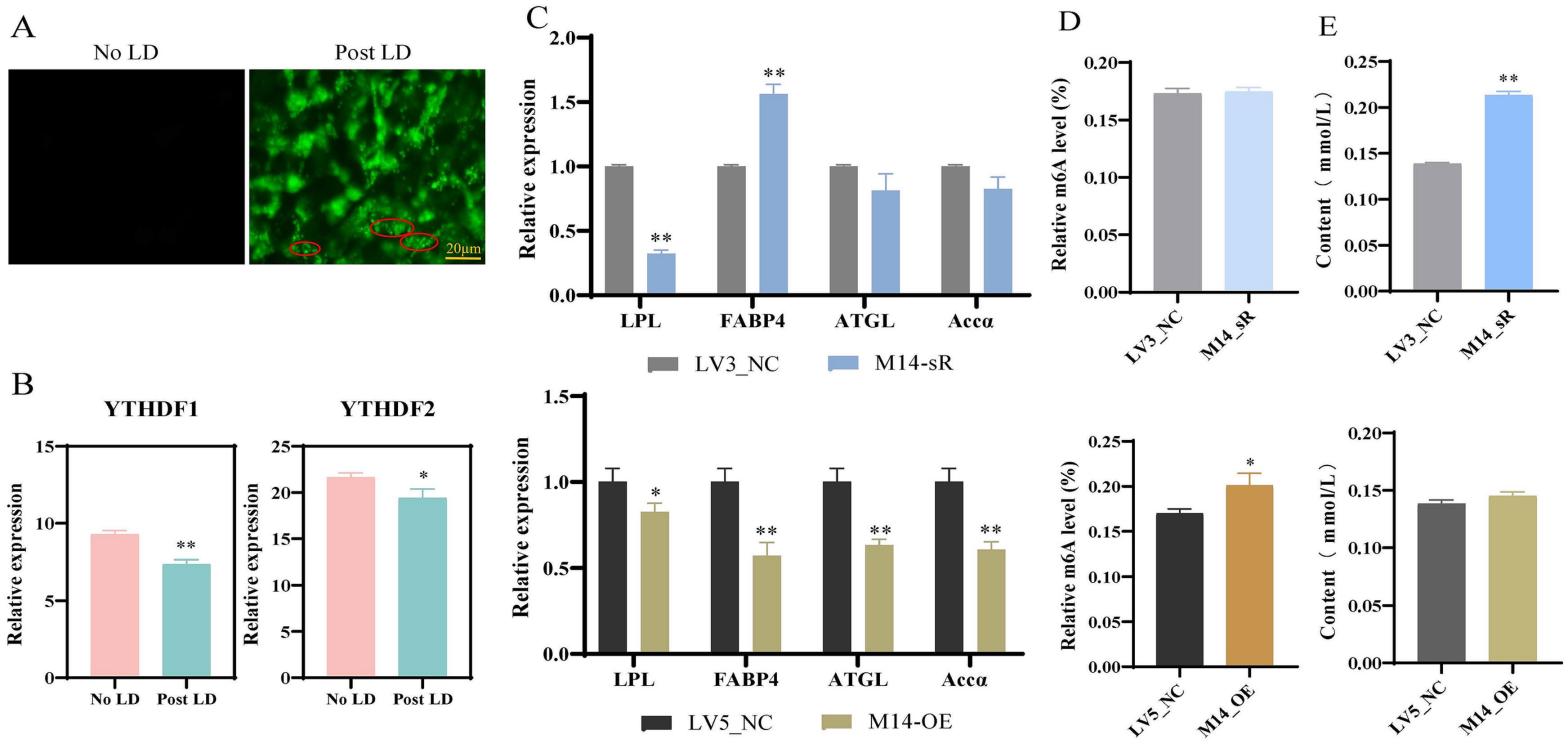
Effect of *METTL14* interference and overexpression on lipid deposition (LD) in primary hepatocytes. **(A)** Bodipy staining detected lipid droplet formation; **(B)** Detection of the relative expression levels of m6A binding protein-related genes; **(C)** Detection of the relative expression levels of lipid metabolism-related genes; **(D)** Detection of the mRNA m6A methylation level; **(E)** Detection of TG content. * *p* < 0.05, ** *p* < 0.01.

Previous findings indicate that *METTL14* is involved in the heat stress response and may modulate heat tolerance through lipid metabolic pathways. However, the precise mechanism by which *METTL14* regulates lipid metabolism remains elusive. To address this, we further explored the molecular mechanisms underlying *METTL14*-mediated lipid metabolic regulation. Bodipy staining revealed a significant increase in green fluorescence intensity and lipid droplet content in primary hepatocytes following induction of lipid deposition compared to the no LD (Figure 3A, No LD: normally cultured cells with no lipid deposition treatment, Post LD: normally cultured cells with lipid deposition treatment). Furthermore, transcriptome sequencing demonstrated a pronounced decrease in the expression levels of the m6A-binding proteins *YTHDF1* and *YTHDF2* under lipid deposition conditions (Figure 3B), supporting the conclusion that *METTL14* is actively involved in regulating lipid deposition.

qRT-PCR analysis showed that compared to that in the LV3_NC, *FABP4* gene expression was significantly upregulated after *METTL14* interference, whereas *LPL* (lipoprotein lipase), *FABP4, ATGL, and Accα* gene expression was significantly decreased after *METTL14* overexpression (Figure 3C), and the m6A methylation level was also significantly increased (Figure 3D). In addition, TG content significantly increased after interference of *METTL14*, and there was no significant difference after overexpressing *METTL14* (Figure 3E). Those results indicated that interference of the *METTL14* gene promoted lipid deposition in primary hepatocytes. Similarly, the expression of *FABP4* and *FAS* was significantly upregulated after *METTL14* interference in preadipocytes, whereas the expression of *LPL, FABP4,* and *ATGL* was significantly downregulated after *METTL14* overexpression (Supplementary Figure S3A), and the m6A methylation levels were significantly decreased after *METTL14* interference (Supplementary Figure S3B); TG content was also significantly increased (Supplementary Figure S3C).

**Figure 4:**
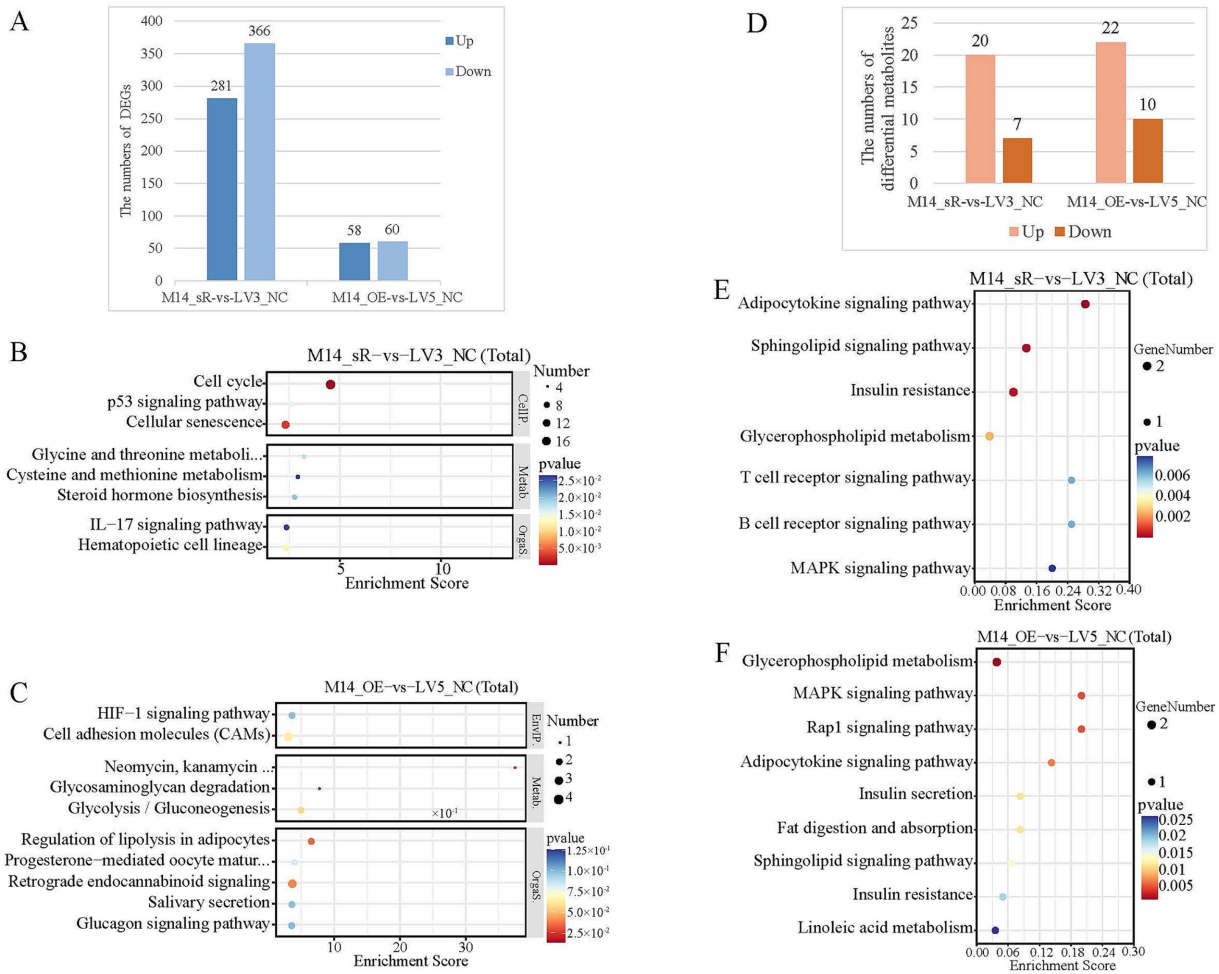
Transcriptomic and metabolic profiles of *METTL14* interference and overexpression in primary hepatocytes. **(A)** Histogram of the number of DEGs for *METTL14* interference and overexpression; **(B)** Bubble diagram of the KEGG pathway enrichment analysis of DEGs in the *METTL14* interference group; **(C)** Bubble diagram of the KEGG pathway enrichment analysis of DEGs in the *METTL14* overexpression group; **(D)** Histogram of differential metabolites quantities for the *METTL14* interference and overexpression; **(E)** Bubble diagram of the KEGG enrichment analysis of differential metabolites in the *METTL14* interference group; **(F)** Bubble diagram of the KEGG enrichment analysis of differential metabolites for the *METTL14* overexpression.

Transcriptome sequencing was performed after interfering and overexpressing *METTL14* in a primary hepatocyte lipid deposition model. Compared with the control group, 281 differentially up-regulated genes and 366 differentially down-regulated genes were identified after interference with *METTL14*, and 58 differentially up-regulated genes and 60 differentially down-regulated genes were identified after overexpression of *METTL14* (Figure 4A). KEGG enrichment analysis of differentially expressed genes revealed that genes were enriched in immune, lipid metabolism and energy metabolism pathways (Figures 4B,C). Primary hepatocytes in the LD group with *METTL14* interference and overexpression were subjected to LC–MS-targeted metabolome analyses. Compared with the LV3_NC group, 20 upregulated and 7 downregulated metabolites were screened after *METTL14* interference (20 positive mode and 7 negative mode), After overexpression of *METTL14*, 22 upregulated and 10 downregulated metabolites were screened (15 positive mode and 17 negative mode, Figure 4D). KEGG enrichment analysis revealed that the differentially metabolites were significantly enriched in adipocytokine signaling pathway and sphingolipid signaling pathway with *METTL14* interference; the differentially metabolites were significantly enriched in glycerophospholipid metabolism, MAPK signaling pathway, fat digestion and absorption with *METTL14* overexpression (Figures 4E,F).

**Figure 5:**
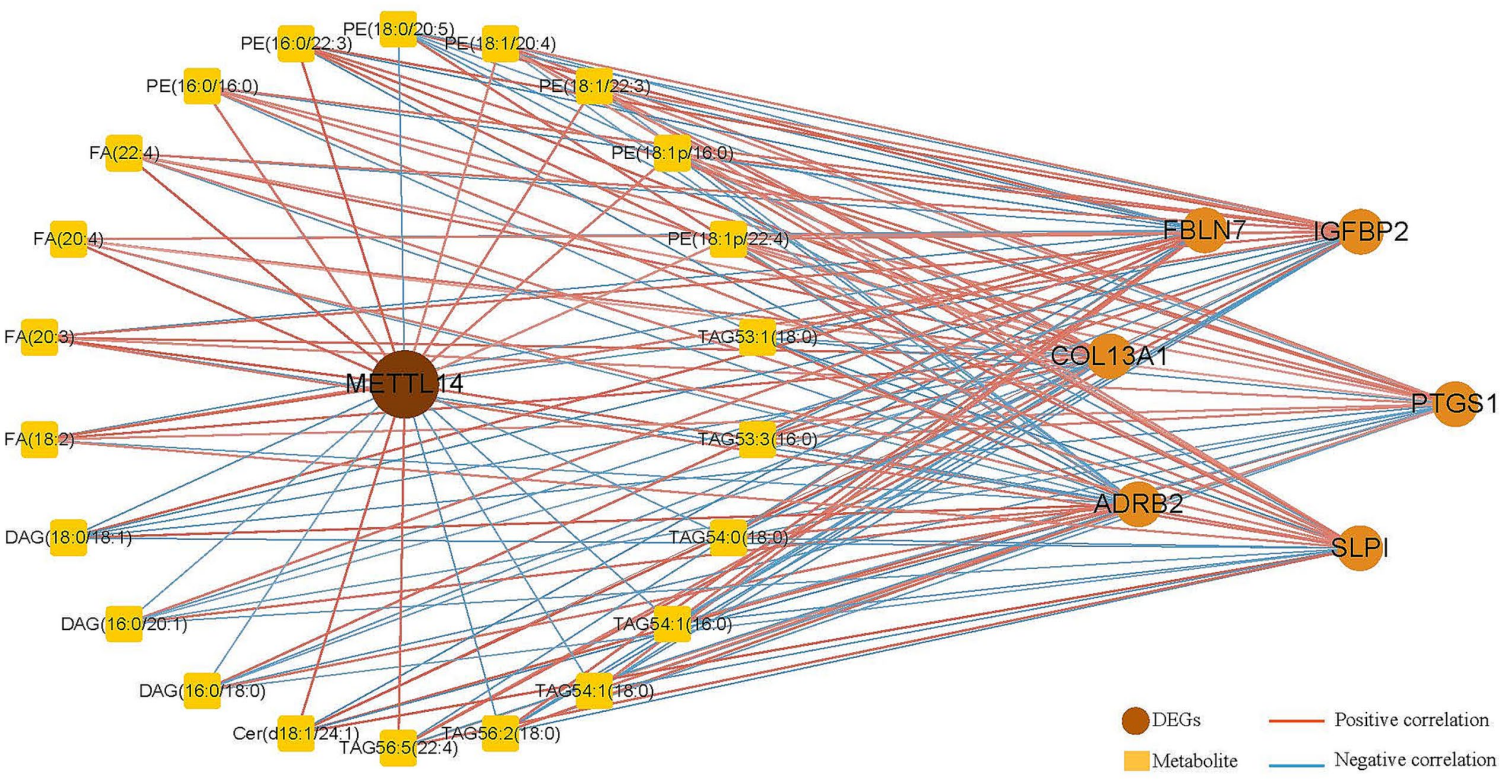
Joint transcriptome and metabolome combined analysis of *METTL14*-related mRNA and metabolites.

Subsequently, integrated transcriptomic and metabolomic analyses were conducted to screen DEGs and differentially abundant metabolites related to lipid metabolism, followed by the construction of a correlation network (Figure 5). Based on the network analysis, DEGs showing strong correlations with *METTL14* were identified, including *FBLN7*, *COL13A1*, *ADRB2*, *SLPI*, *PTGS1*, and *IGFBP2*. In addition, several differentially abundant metabolites highly associated with *METTL14* were also discerned, such as phosphatidylethanolamine (PE), fatty acids (FAs), diacylglycerol (DAG), triacylglycerol (TAG), and ceramide (Cer).

### 3.4 The molecular mechanism of *METTL14* regulating heat stress in Hu sheep primary hepatocytes and preadipocytes through lipid metabolism

**Figure 6:**
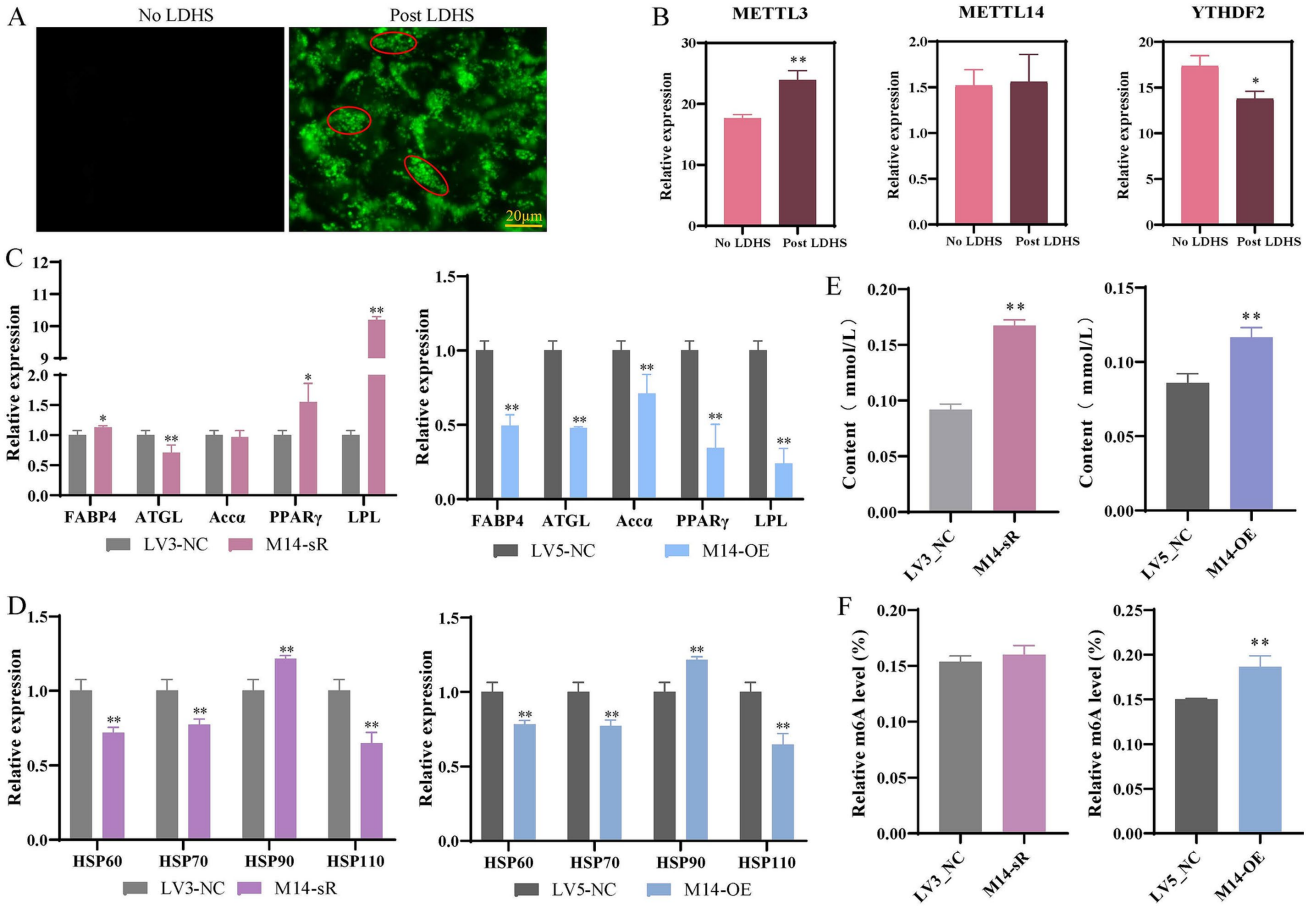
Effects of interference and overexpression of *METTL14* on lipid deposition heat stress (LDHS) of in primary hepatocytes. **(A)** Bodipy staining detected lipid droplet formation; **(B)** Expression of *METTL3*, *METTL14*, and *YTHDF2* in the RNA-seq of primary hepatocytes; **(C)** Detection of lipid metabolism-related gene expression levels; **(D)** Detection of expression levels of heat stress-related genes; **(E)** Detection of TG content; **(F)** Detection of m6A methylation level. * *p* < 0.05, ** *p* < 0.01.

Bodipy staining revealed a significant increase in green fluorescence intensity in primary hepatocytes subjected to combined lipid deposition and heat stress compared to the no LDHS (Figure 6A, No LDHS: normally cultured cells with no lipid deposition heat stress treatment, Post LD: normally cultured cells with lipid deposition heat stress treatment). The lipid droplet content was markedly higher than that induced by lipid deposition alone (as shown in Figure 3A), further supporting the role of heat stress in promoting lipid accumulation. Transcriptome analysis indicated that under heat stress conditions, *METTL3* expression was significantly upregulated following lipid deposition, while *METTL14* expression showed a non-significant increasing trend. In contrast, *YTHDF2* expression was significantly downregulated (Figure 6B).

In comparison to the LV3_NC group, the expression of lipid metabolism-related genes, specifically *FABP4*, *PPARγ* and *LPL*, was significantly elevated following the interference with *METTL14*. Conversely, the expression levels of *FABP4*, *ATGL*, *Accα*, *PPARγ*, and *LPL* were significantly reduced upon the overexpression of *METTL14* (Figure 6C). Additionally, the expression of heat shock-related genes, including *HSP60*, *HSP70*, and *HSP110*, significantly decreased after the overexpression of *METTL14* (Figure 6D). Notably, TG content increased significantly following both interference and overexpression of *METTL14* (Figure 6E). Furthermore, the level of m6A methylation was significantly enhanced after the overexpression of *METTL14* (Figure 6F). These findings suggest that the overexpression of the *METTL14* gene may reduce the expression of heat shock genes by repressing the expression of lipid metabolism-related genes in an m6A-dependent manner. In preadipocytes, the expression of lipid metabolism-related genes *FABP4*, *Accα* and *LPL* was significantly increased following interference with *METTL14*, while the expression of *ATGL*, *Accα*, and *LPL* was significantly diminished after *METTL14* overexpression (Supplementary Figure S4A). Additionally, the expression of heat shock-related genes *HSP70* and *HSP90* significantly decreased after *METTL14* overexpression (Supplementary Figure S4B). TG content was significantly elevated and the m6A methylation level was also significantly increased following interference with *METTL14*. In contrast, TG content significantly decreased after the overexpression of *METTL14*, mirroring the results observed in primary hepatocytes (Supplementary Figures S4C,D).

**Figure 7:**
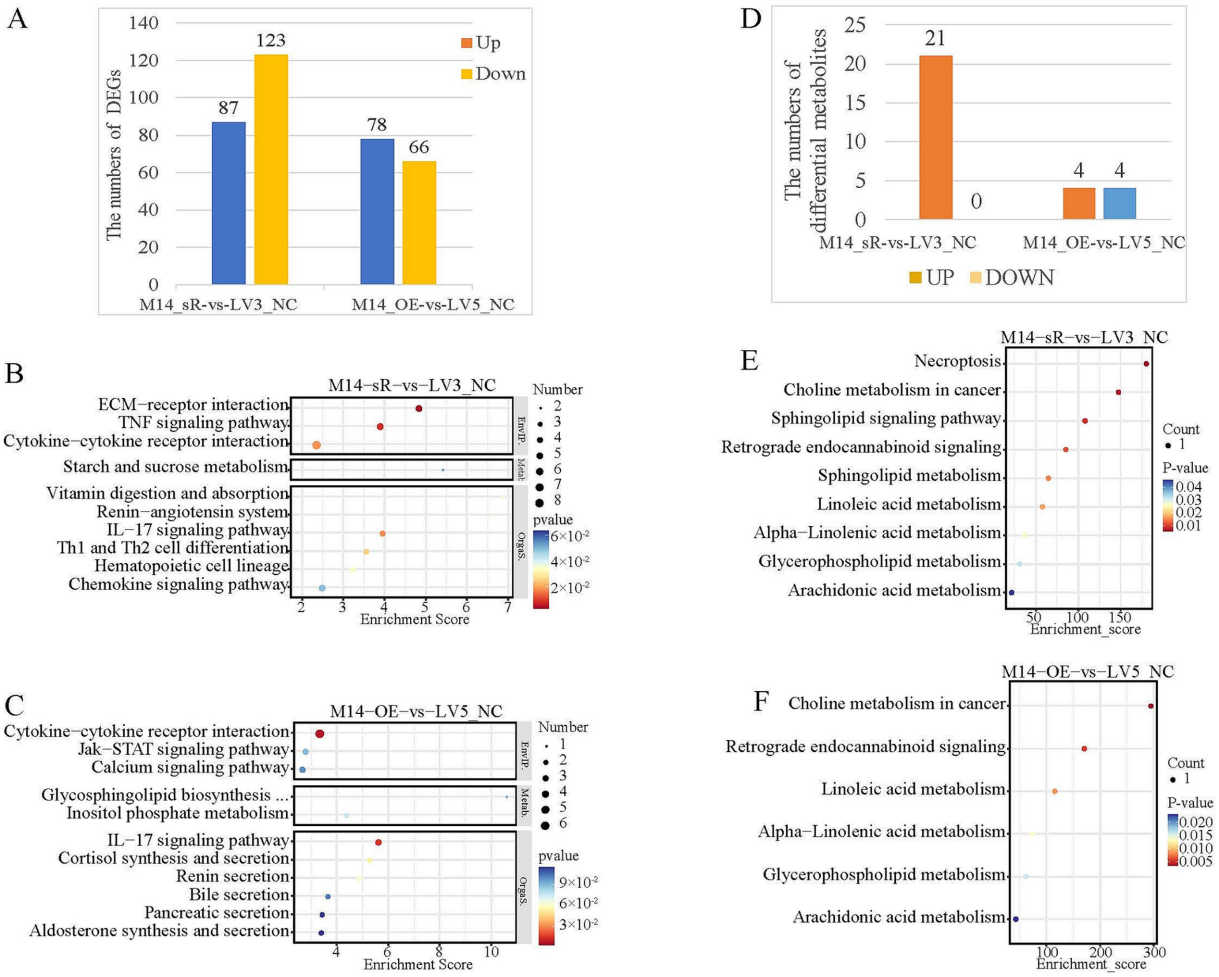
Transcriptome and metabolomics analysis after interference and overexpression of *METTL14* in primary hepatocyte LDHS model. **(A)** Histogram of the number of differentially expressed genes; **(B)** KEGG enrichment analysis of DEGs after interference with *METTL14*; **(C)** KEGG enrichment analysis of DEGs after overexpression of *METTL14*; **(D)** Histogram of differential metabolites quantities; **(E)** KEGG enrichment analysis of differential metabolites after interference with *METTL14*; **(F)** KEGG enrichment analysis of differential metabolites after overexpression of *METTL14*.

Transcriptome sequencing was conducted following the interference and overexpression of *METTL14* in a heat stress model of primary hepatocellular fat deposition. Compared to the control group, interference with *METTL14* resulted in the identification of 87 differentially up-regulated genes and 123 differentially down-regulated genes. In contrast, overexpression of *METTL14* led to the identification of 78 differentially up-regulated genes and 66 differentially down-regulated genes (Figure 7A). KEGG enrichment analysis of the differentially expressed genes indicated that, after *METTL14* interference, the genes were primarily enriched in the TNF signaling pathway, as well as the renin-angiotensin and IL-17 pathways (Figure 7B). Conversely, following *METTL14* overexpression, the genes were significantly enriched in fat digestion and absorption pathway (Figure 7C). Additionally, LC–MS metabolomics sequencing was performed after the interference and overexpression of *METTL14* in the same heat stress model of primary hepatocyte lipid deposition. Compared to the control group, 21 differentially up-regulated metabolites were identified following *METTL14* interference (comprising 12 positive mode and 9 negative mode). In contrast, after *METTL14* overexpression, 4 differentially up-regulated metabolites and 4 differentially down-regulated metabolites were identified (consisting of 4 positive mode and 4 negative mode, Figure 7D). KEGG enrichment analyses demonstrated that the differentially regulated metabolites following *METTL14* interference or overexpression were enriched in lipid metabolism-related pathways, including sphingolipid metabolism, linoleic acid metabolism, and arachidonic acid metabolism (Figures 7E, F).

**Figure 8:**
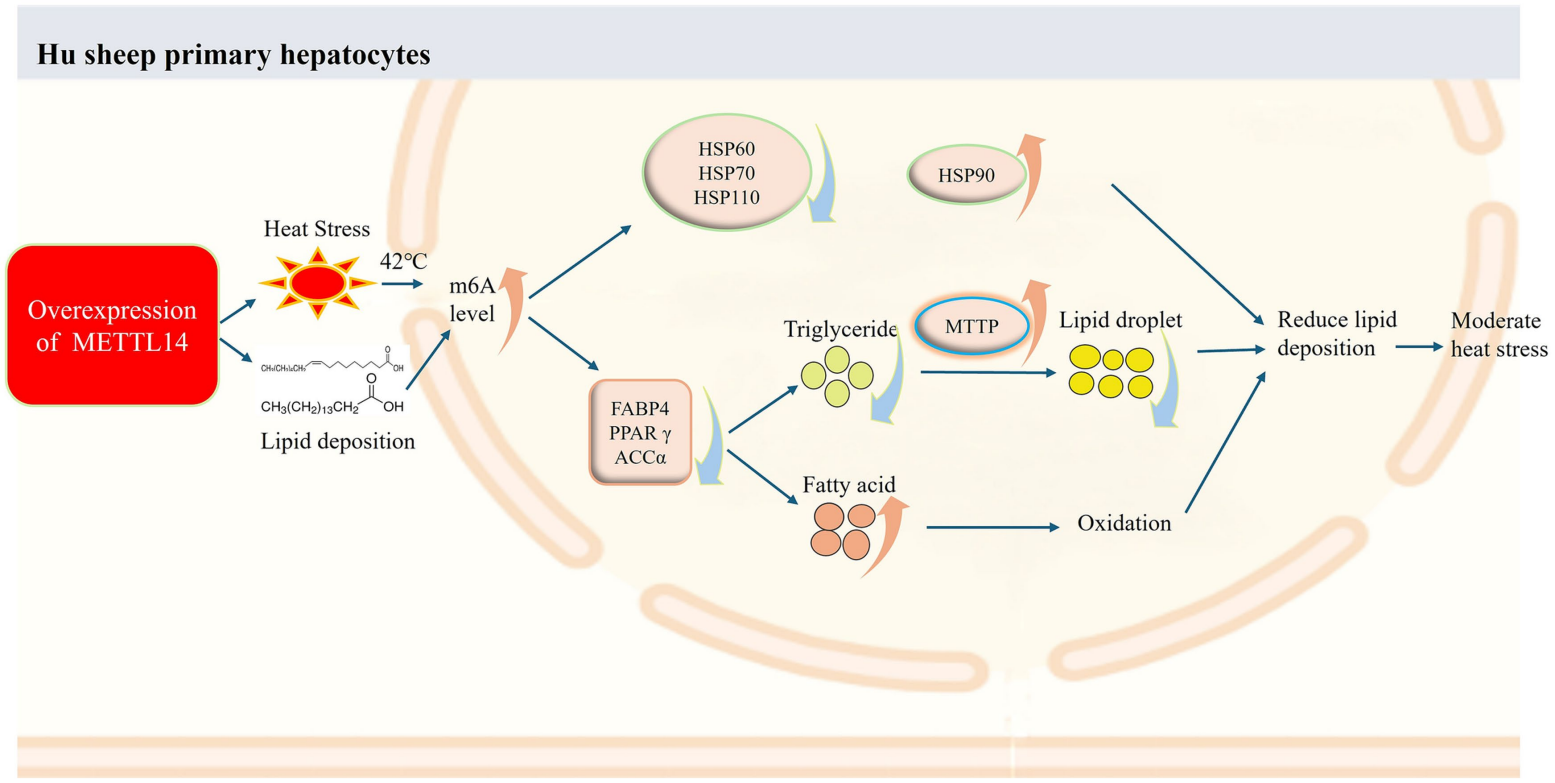
Molecular mechanism of *METTL14*-mediated attenuation of heat stress in Hu sheep through enhanced fatty acid oxidation and reduced hepatic triglyceride accumulation.

Based on the above findings, we propose a mechanistic model illustrating how *METTL14*-mediated metabolic reprogramming alleviates heat stress in Hu sheep (Figure 8). Under conditions mimicking adipogenic heat stress, overexpression of *METTL14* enhances m6A methylation levels in Hu sheep hepatocytes. This leads to the downregulation of heat shock genes (*HSP60*, *HSP70*, *HSP110*) and lipogenic genes (*FABP4*, *PPAR*γ, *ACC*α). Concurrently, upregulation of MTTP facilitates triglyceride export, thereby reducing intracellular triglyceride content. In parallel, fatty acid oxidation is promoted. Together, these metabolic changes attenuate lipid accumulation and contribute to the amelioration of heat stress in Hu sheep.

## 4 Discussion

Heat stress, exacerbated by global warming, represents a critical challenge to the global livestock sector. Ruminants, growing pigs, and poultry are especially vulnerable due to their high metabolic rates, rapid growth, intensive production output, and thermosensitivity (28). Heat stress negatively impacts voluntary feed intake (29, 30), compromises the antioxidant defense system (31), disrupts mitochondrial function, and alters heat shock protein expression (31–33). It induces oxidative stress by disturbing free radical homeostasis, reprograms the metabolism of proteins, lipids, and energy (34), and thereby impairs overall productivity, reproductive performance, and animal health. These effects collectively lead to reduced efficiency in livestock production systems.

As a core component of the methyltransferase complex (composed of METTL3, METTL14, and WTAP), *METTL14* plays a central role in catalyzing m6A modifications on RNA, thereby modulating mRNA stability, translation efficiency, and splicing processes (35, 36). The m6A modification serves as a critical regulator of gene expression by affecting mRNA decay, subcellular localization, and translational dynamics. Previous studies have demonstrated that heat stress induces significant upregulation of *METTL14* expression, which correlates strongly with the induction of heat shock protein. For example, in a sheep model, heat stress resulted in elevated *METTL14* mRNA and protein levels, along with increased expression of *HSP70*, *HSP90,* and *HSP110* (11), suggesting that *METTL14* may regulate HSP transcription or translation via m6A-dependent mechanisms to enhance cellular thermotolerance. To further investigate this hypothesis, the current study overexpressed *METTL14* in hepatocytes and adipocytes derived from Hu sheep and observed a marked downregulation of heat shock-related genes. These findings provide functional evidence for the regulatory role of *METTL14* in HSP expression. Moreover, *METTL14* contributes to the cellular heat stress response through interactions with other m6A regulatory proteins. For instance, in sheep subjected to heat stress, *METTL14* acts in concert with *METTL3*, *WTAP*, and *FTO* to regulate m6A methylation levels, thereby facilitating cellular adaptation to thermal challenge (11). Conversely, deficiency or functional impairment of *METTL14* has been shown to increase cellular susceptibility to heat stress, compromising survival and tissue homeostasis (37).

Heat stress not only modulates the expression of heat shock proteins but also significantly influences genes involved in lipid metabolism. In a porcine model, maternal heat stress was found to markedly upregulate *METTL14* expression in the liver and abdominal adipose tissue of offspring, concomitant with increased expression of key lipogenic genes including *DGAT1*, *SREBP-1c*, and *PPARγ* (18). These findings imply that *METTL14* may participate in the regulation of lipid metabolism-related genes via m6A RNA methylation, thereby influencing adipogenesis under heat stress conditions. To further investigate the regulatory function of *METTL14* in the heat stress response of Hu sheep, this study established an ex vivo cell model by exposing cells to 42 °C heat stress combined with lipid accumulation induction, effectively mimicking *in vivo* physiological alterations under thermal challenge (38). Subsequent knockdown and overexpression experiments revealed that *METTL14* overexpression led to significant alterations in metabolites closely associated with lipid metabolism, specifically triacylglycerol (TAG) and fatty acids (FA). Additionally, the expression of *MTTP*, a gene critically involved in triglyceride transport (39), was examined. Previous studies indicate that *MTTP*, a member of the large lipid transfer protein superfamily, plays an essential role in the assembly of ApoB-containing very-low-density lipoproteins (VLDL) and facilitates triglyceride (TG) trafficking. Pharmacological inhibition of *MTTP* in mice resulted in pronounced reductions in plasma total cholesterol and TG levels (40). Similarly, liver-specific *MTTP* knockout mice demonstrated markedly decreased plasma cholesterol and TG, accompanied by severe hepatic steatosis and lipid accumulation (41) In the current study, elevated *MTTP* expression was observed to promote the export of triglycerides from hepatocytes and enhance intracellular fatty acid utilization, thereby attenuating lipid droplet accumulation and mitigating heat stress-induced cellular damage. As a core component of the m6A methylation machinery, research on *METTL14’s* role in heat stress regulation offers novel perspectives for both animal science and biomedical applications. For example, targeted modulation of *METTL14* expression or activity may represent a promising strategy to enhance thermotolerance and productivity in livestock (12, 18).

Given the substantial adverse effects of heat stress on animal production, the livestock industry increasingly relies on integrated management strategies that encompass both adaptation to climate change pressures and mitigation of environmental impacts. Adaptation approaches include the selection of thermotolerant breeds, optimization of water provisioning, and enhancement of forage diversity. Mitigation strategies involve nutritional interventions—such as refined feeding regimens and targeted nutrient supplementation—modulation of rumen function, and the implementation of physical cooling measures including shade structures, improved housing, ventilation fans, and sprinkler systems (6). The formulation of effective nutritional interventions necessitates a mechanistic understanding of heat stress responses. Current research has made considerable progress in elucidating the underlying physiological and molecular mechanisms. For instance, melatonin has been demonstrated to ameliorate heat stress-induced spermatogenic impairment in dairy goats, primarily through modulation of the gut microbiota and subsequent suppression of excessive arachidonic acid biosynthesis in testicular tissue (42). Dietary supplementation with selenomethionine enhances hepatic selenium retention and antioxidant capacity in broilers, thereby mitigating mitochondrial dysfunction and aberrations in the tricarboxylic acid (TCA) cycle, which in turn restores hepatic triglyceride and glycogen homeostasis (43). Another study reported that betaine supplementation reduced serum triglyceride levels and increased non-esterified fatty acid concentrations in broilers (44). Based on the findings of Zhu et al. (45, 46), it is hypothesized that dietary supplementation with methyl donors—such as trimethylglycine—or modulation via methylation inhibitors (e.g., cycloleucine) may similarly attenuate heat stress responses in Hu sheep, whether at the hepatocyte level or *in vivo*. Nevertheless, the precise regulatory mechanisms warrant further in-depth investigation.

## 5 Conclusion

*METTL14* is a key regulator coordinating the heat stress response and adipogenesis. It reduces the expression of heat shock genes (*HSP60*, *HSP70*, *HSP110*) and key lipogenic genes (*FABP4*, *PPARγ*, *Accα*) by elevating m6A RNA methylation, while concurrently upregulating the lipid export - MTTP gene to stimulate triglyceride efflux. Together with increased fatty acid oxidation, these alterations collectively diminish lipid accumulation in hepatocytes. This coordinated response constitutes an adaptive metabolic remodeling strategy whereby the organism inhibits lipid synthesis and promotes lipid breakdown and export under heat stress. Consequently, this pathway alleviates lip toxicity, maintains metabolic homeostasis, and enhances overall stress tolerance. *METTL14* may represent a potential target for the molecular breeding of heat-tolerant varieties, offering a theoretical basis for developing new cultivars with improved thermotolerance.

## Data Availability

The data presented in this study are deposited in the SRA repository, accession number: PRJNA988822, PRJNA1050989, and PRJNA1178813.

## Footnotes

1
http://www.genome.jp/kegg/

2
https://www.omicshare.com/tools/Home/Soft/enrich_circle
