# Supplementary material for: METTL14 alleviates heat stress in Hu sheep involves enhancing fatty acid oxidation while reducing lipid deposition
Source: Front Vet Sci. 2026 Jan 19;12:1732947. doi: 10.3389/fvets.2025.1732947 (PMC12862939; doi:10.3389/fvets.2025.1732947)
Supplement: Supplementary file 1 [file Data_Sheet_1.docx]

Supplementary Material

# Supplementary Figures and Tables

## Supplementary Figures


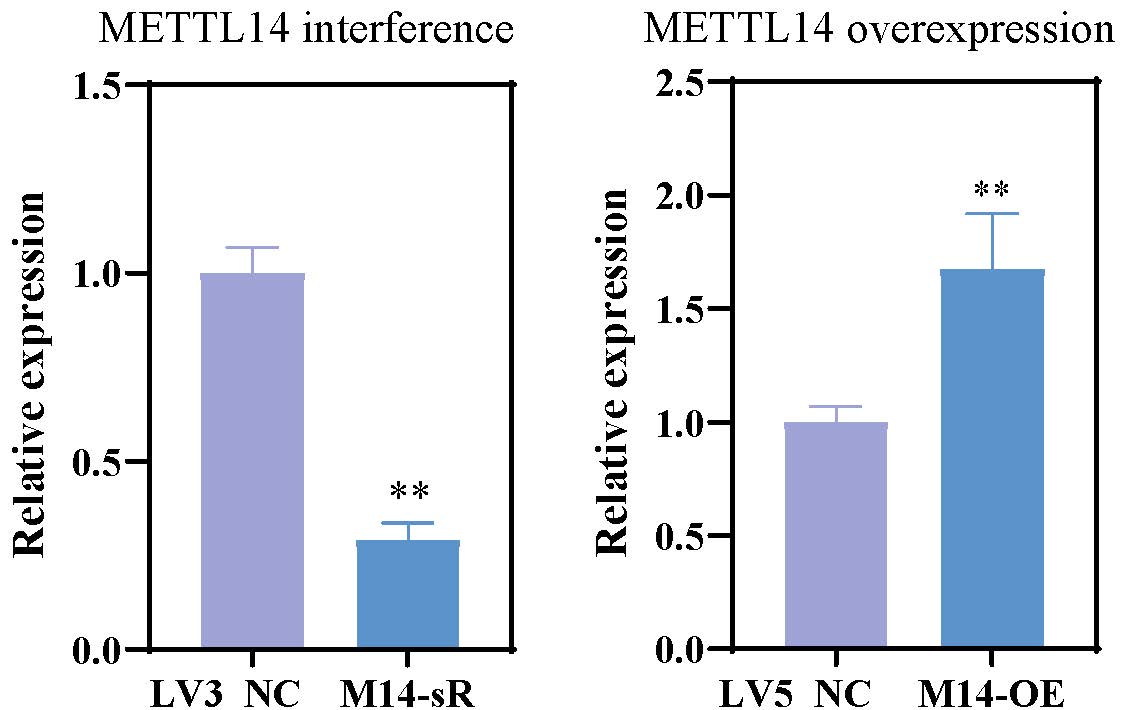


Figure S1 Interference and overexpression efficiency detection of *METTL14* gene. * *p* < 0.05, ** *p* < 0.01.


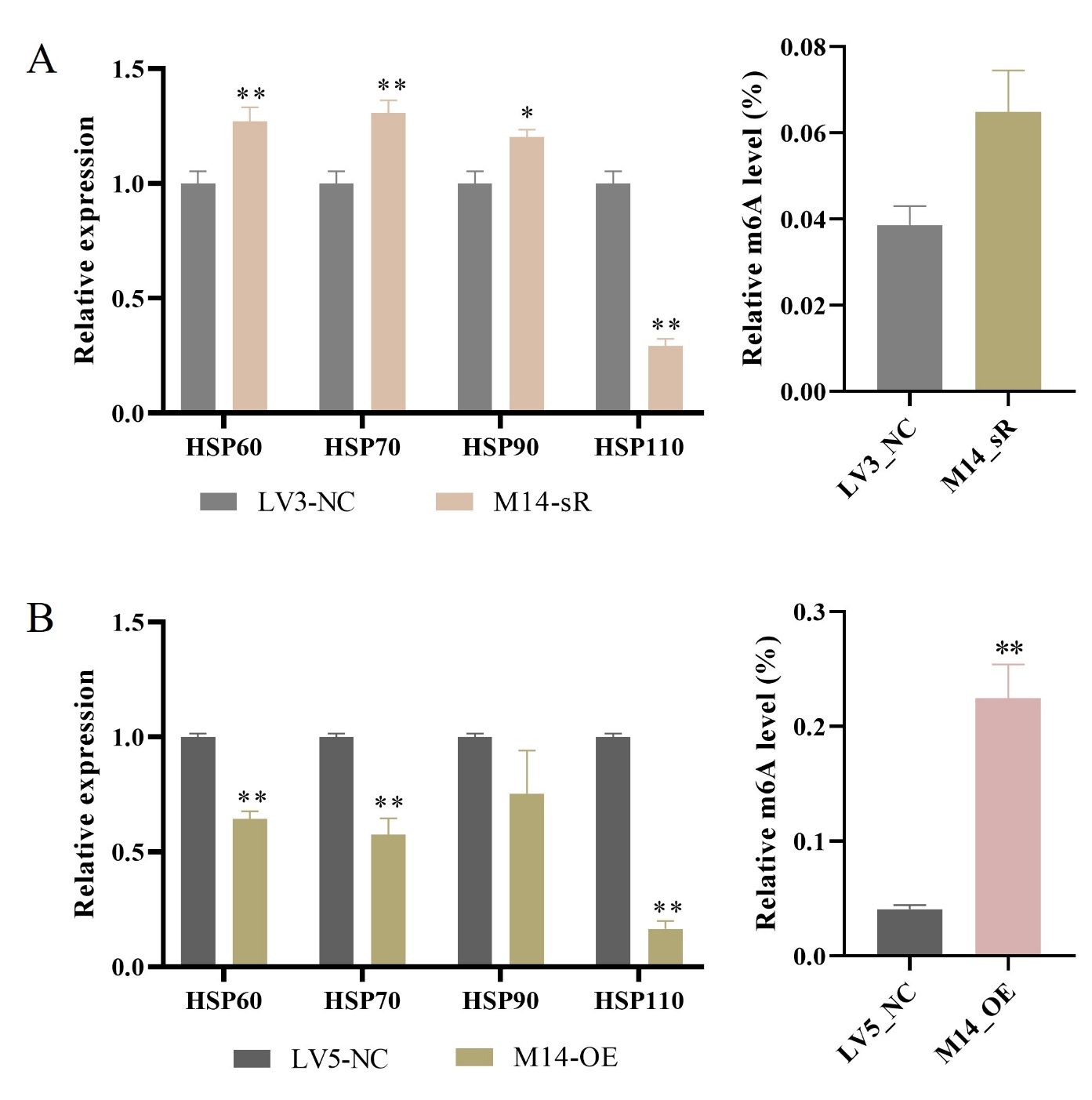


Figure S2 Detection of heat shock gene expression changes and m6A methylation levels of *METTL14* interference and overexpression in preadipocytes. (A) RT-qPCR analysis of heat shock gene expression changes and mRNA m6A methylation quantification of *METTL14* interference; (B) RT-qPCR analysis of heat shock gene expression changes and mRNA m6A methylation quantification of *METTL14* overexpression. * *p* < 0.05, ** *p* < 0.01.


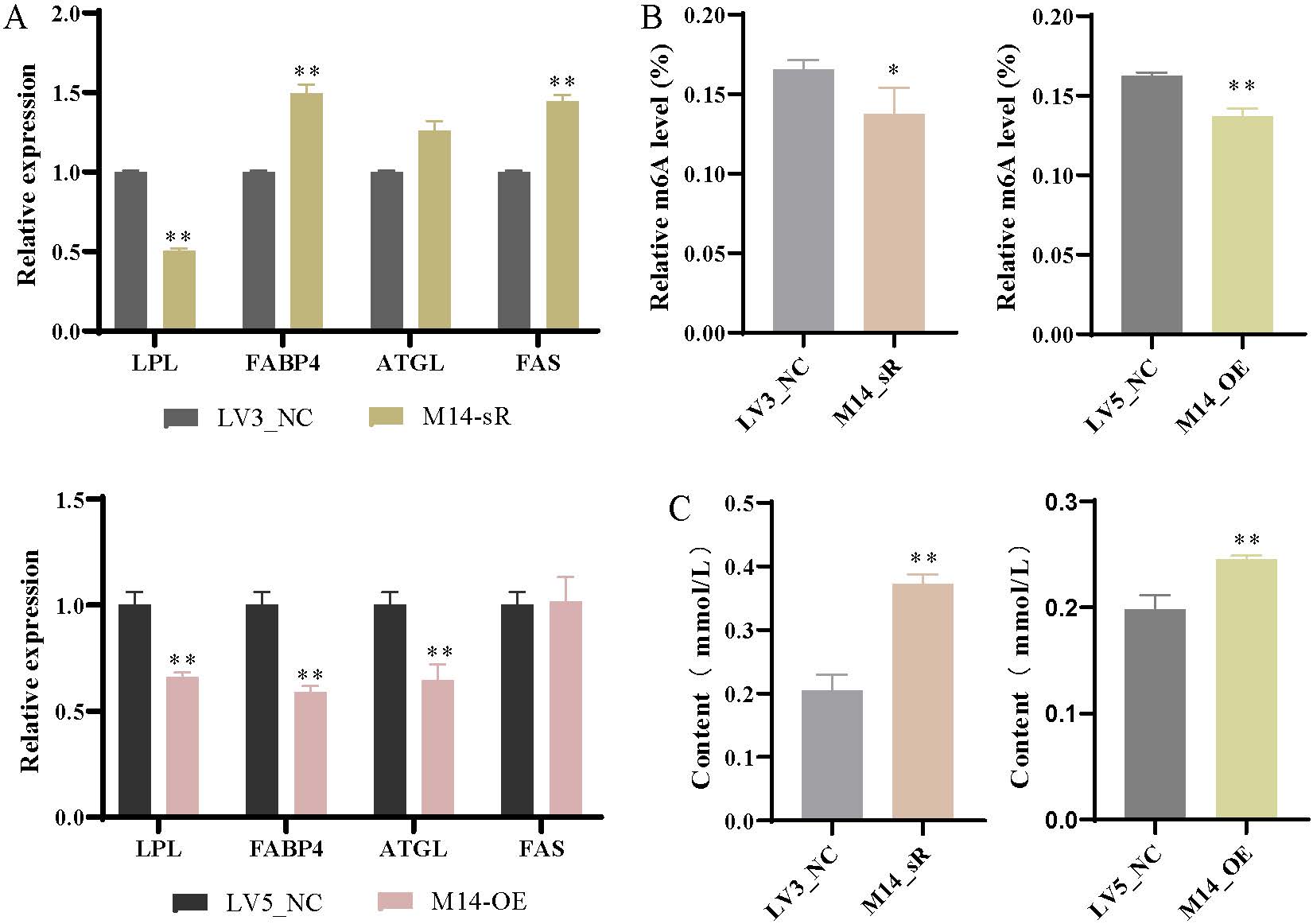


Figure S3 Effect of interference and overexpression of *METTL14* on lipid deposition in preadipocytes (A) RT-qPCR was used to detect the relative expression levels of lipid metabolism-related genes; (B) m6A methylation level detection; (C) Detection of triglyceride content. * *p* < 0.05, ** *p* < 0.01.


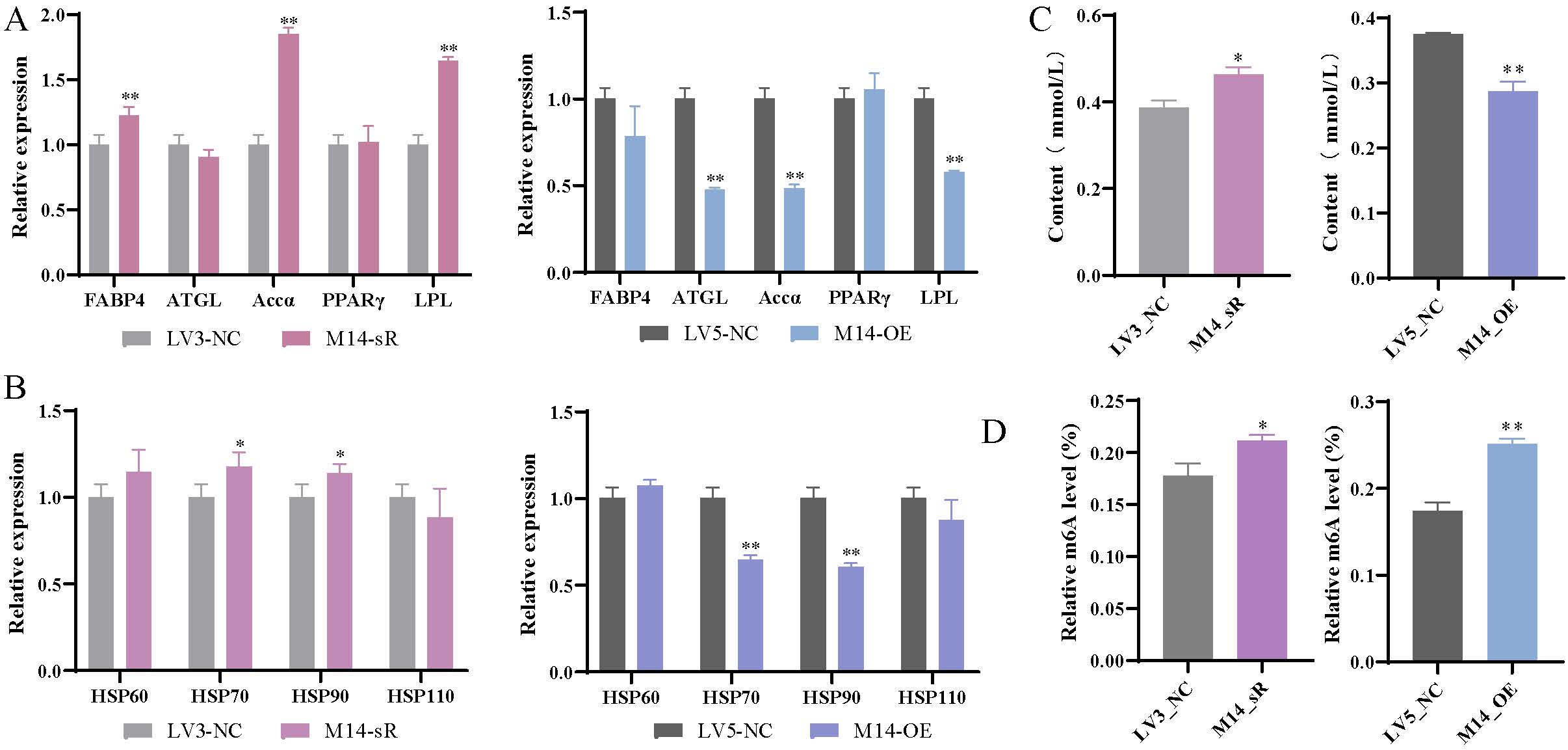


Figure S4 Detection of gene expression changes and m6A methylation levels of *METTL14* interference and overexpression in preadipocytes. (A) RT-qPCR was used to detect the relative expression levels of lipid metabolism-related genes; (B) RT-qPCR analysis of heat shock gene expression change; (C) Detection of triglyceride content; (D) m6A methylation level detection. * *p* < 0.05, ** *p* < 0.01.

## Supplementary Tables

Table S1 Primer sequence information of *METTL14* gene

| B9712-1 | AGGGTTCCAAGCTTAAGCGGCCGCGCCACCATGCGCGTGCG |
| --- | --- |
| B9712-2 | GGTGGCAGGTGAGGAGACGGAAAAACGGGGAGAAAGGGACGGGGAACGCGGCGCACGCGCATGGT |
| B9712-3 | TCTCCTCACCTGCCACCCGGCAGCCCTTCCTGGTCTTGCGACTTTAATCCCAGGACCTCTCTACC |
| B9712-4 | CGCAAGGACCACAACATGAAAGGGGAACGATTCGGGGTAGAGAGGGTAGAGAGGTCCTGGGATTA |
| B9712-5 | TCATGTTGTGGTCCTTGCGGTGGTGCTAAGCCGACCGCCACGCCTGGTCTTCAGCATCCTGTGTG |
| B9712-6 | TTTGTATCCTCATCACTGAGGATGCCAGACCCCTGGCGGGCATTCCACACAGGATGCTGAAGACC |
| B9712-7 | ATCCTCAGTGATGAGGATACAAAACTTTGGTGTTTGTGTTTGGGAGCTGAAAGTGCTGACAGCAT |
| B9712-8 | TCAGCAATTTCTCTCTGCTCATCTTTGCTATTTAACACAGCACCAATGCTGTCAGCACTTTCAGC |
| B9712-9 | TGAGCAGAGAGAAATTGCTGAAACAAGAGAAACTTGCAGGGCTTCCTATGATACCTCTGCTCCAA |
| B9712-10 | TCTTCATCTGTCTCTCCTTCATCCTGATACTTACGTTTTGCATTTGGAGCAGAGGTATCATAGGA |
| B9712-11 | GATGAAGGAGAGACAGATGAAGACAAGATGGAAGAATATAAGGATGAATTAGAAATGCAACAGGA |
| B9712-12 | AATCTTTATAAATCTCTTCTTCATATGGCAAATTCTCTTCCTCCTGTTGCATTTCTAATTCATCC |
| B9712-13 | GCCATATGAAGAAGAGATTTATAAAGATTCTAGTACTTTTCTTAAGGGAACACAGAGTTTAAATC |
| B9712-14 | TGTCCAGTGTCTACAAAATGTTGGCAGTAATCATTATGGGGATTTAAACTCTGTGTTCCCTTAAG |
| B9712-15 | CAACATTTTGTAGACACTGGACATAGACCCCAGAATTTCATCAGGGATGTAGGTTTGGCCGACAG |
| B9712-16 | ATCCTTCAGTCTGATGAGCTCCCGCAGTTTAGGGTATTCTTCAAATCTGTCGGCCAAACCTACAT |
| B9712-17 | GAGCTCATCAGACTGAAGGATGAGTTAATAGCTAAATCTAACACTCCTCCTATGTACTTACAAGC |
| B9712-18 | AATTTGGGTGTCAGTTCTCTGATGTCAAAGGCTTCTATATCTGCTTGTAAGTACATAGGAGGAGT |
| B9712-19 | TCAGAGAACTGACACCCAAATTTGATGTGATTCTTCTTGAACCACCTTTAGAAGAATATTACAGA |
| B9712-20 | CCAAGTCCAGCATTTTTCATTAGCAGTGATGCCGGTCTCTCTGTAATATTCTTCTAAAGGTGGTT |
| B9712-21 | CTAATGAAAAATGCTGGACTTGGGATGATATTATGAAGTTAGAAATTGATGAGATTGCAGCACCT |
| B9712-22 | TCCAATCCTTCCCCAGAACCACACCAGAGAAAAATAAATGATCGAGGTGCTGCAATCTCATCAAT |
| B9712-23 | GGTTCTGGGGAAGGATTGGACCTTGGAAGAGTGTGTTTACGCAAGTGGGGTTACAGAAGATGTGA |
| B9712-24 | TCTTCCCAGGATTGTTTTTATTGGTTTTAATCCAACAAATATCTTCACATCTTCTGTAACCCCAC |
| B9712-25 | CCAATAAAAACAATCCTGGGAAGACTAAGACTTTAGATCCAAAGGCCGTCTTCCAGAGAACAAAG |
| B9712-26 | TGTGCTACGCTTAACAGTTCCTTTGATCCCCATAAGGCAGTGTTCCTTTGTTCTCTGGAAGACGG |
| B9712-27 | GGAACTGTTAAGCGTAGCACAGACGGGGACTTCATTCATGCTAATGTTGACATTGACTTGATTAT |
| B9712-28 | ACAGGTTTTTCTATATTGCCAATTTCAGGTTCTTCTGTGATAATCAAGTCAATGTCAACATTAGC |
| B9712-29 | AAATTGGCAATATAGAAAAACCTGTAGAAATTTTTCATATAATTGAACATTTTTGTCTTGGTAGA |
| B9712-30 | GTCGAATTGTACTATCTCTTCCAAACAGATGAAGGCGTCTTCTACCAAGACAAAAATGTTCAATT |
| B9712-31 | TTTGGAAGAGATAGTACAATTCGACCAGGGTGGCTTACAGTTGGACCAACTCTTACAAATAGTAA |
| B9712-32 | CGGGGCACTGAAGTAGGATGCATACGTTTCTGCATTGTAGTTACTATTTGTAAGAGTTGGTCCAA |
| B9712-33 | TCCTACTTCAGTGCCCCGAATTCCTATTTGACTGGATGTACAGAGGAAATTGAGAGACTTCGACC |
| B9712-34 | TCCACCTCCCCGATCAGATTTAGATTTGGGAGGAGGTGATTTTGGTCGAAGTCTCTCAATTTCCT |
| B9712-35 | TCTGATCGGGGAGGTGGAGCTCCCAGAGGAGGCGGAAGAGGTGGCACTTCTGCTGGCCGTGGGCG |
| B9712-36 | CCCCCCCTGAAGCCACCTCTTTCTCCTCGGAAGTTAGATCGATTTCTCTCTCGCCCACGGCCAGC |
| B9712-37 | GTGGCTTCAGGGGGGGCCGTGGAGGAGCACACAGAGGTGGCTTTCCACCTCGATAGGGATCCGAC |
| B9712-38 | ATCAGTAGAGAGTGTCGGATCCCTATCGAGGT |
